# A Shared Control Approach to Robot-Assisted Cataract Surgery Training for Novice Surgeons

**DOI:** 10.3390/s25165165

**Published:** 2025-08-20

**Authors:** Balint Varga, Michael Poncelet

**Affiliations:** Institute of Control Systems (IRS), Karlsruhe Institute of Technology (KIT), 76131 Karlsruhe, Germany

**Keywords:** haptic shared control, teleoperated surgery, robot-assisted surgery, human factors, virtual fixtures

## Abstract

This paper proposes a novel virtual-fixtures-based shared control concept for eye surgery systems focusing on cataract procedures, one of the most common ophthalmic surgeries. Current research on haptic force feedback aims to enhance manipulation capabilities by integrating teleoperated medical robots. Our proposed concept utilizes teleoperated medical robots to improve the training of young surgeons by providing haptic feedback during cataract operations based on geometrical virtual fixtures. The core novelty of our concept is the active guidance to the incision point generated directly from the geometrical representation of the virtual fixtures, and, therefore, it is computationally efficient. Furthermore, novel virtual fixtures are introduced for the posterior corneal surface of the eye during the cataract operation. The concept is tested in a human-in-the-loop pilot study, where non-medical engineering students participated. The results indicate that the proposed shared control system is helpful for the test subjects. Therefore, the inclusion of the proposed concept can be beneficial for the training of non-experienced surgeons.

## 1. Introduction

In the field of human–machine systems, creating seamless interactions between humans and technology is crucial, particularly as automation becomes increasingly prevalent in our daily lives; see, e.g., [1,2]. Adaptive human–machine systems are designed to provide intuitive and responsive control, improving user experience and precision in complex tasks [3,4]. Surgical applications represent a field where these systems demonstrate significant value [5,6]. Achieving full automation in surgery is challenging due to the intricate tasks requiring exceptional precision and adaptability, as highlighted in [7,8]. The approval and registration of these systems can be critical due to the stringent safety, performance, and regulatory standards that must be met to ensure patient safety.

However, the integration of teleoperated medical robots has the potential to increase surgical precision (teleoperated surgery and robot-assisted surgical systems are overlapping areas of research. In this paper, robot assistance is implemented through teleoperation, and, as such, both terms are used interchangeably). Robot-assisted surgical systems help surgeons to perform their tasks more efficiently and safely. Features such as virtual fixtures provide tool positioning constraints to prevent damage to sensitive tissues [9].

Medical robots are utilized for various surgeries in clinical practice, with ongoing research focused on their integration into ophthalmic surgery. Robotic assistance offers several advantages, including greater precision, fewer complications, and faster healing times. This technology is particularly beneficial in surgical procedures requiring high accuracy in areas with limited visibility and space, such as cataract surgery [10]. In developing countries, where access to highly experienced surgeons may be limited, robotic assistance can empower less experienced surgeons to perform complex surgeries effectively and can help to increase the cataract surgery rate [9].

One significant focus within various surgical procedures is cataract removal, a common clinical condition primarily treated surgically. The most common type, senile cataracts, leads to age-related lens opacity. In Germany, lenticular opacity affects 50% of individuals aged 60 and above, rising to 90% for those over 75 [11]. If left untreated, cataracts can ultimately result in blindness. Globally, cataracts account for one-third of the 35 million blind individuals [9], and approximately 20 million cataract surgeries are performed yearly [12]. The rising life expectancy in industrialized nations is leading to an increased need for primary and secondary cataract surgeries [11]. Most cataract surgeries are still performed manually, without any robotic assistance.

**Figure 1:**
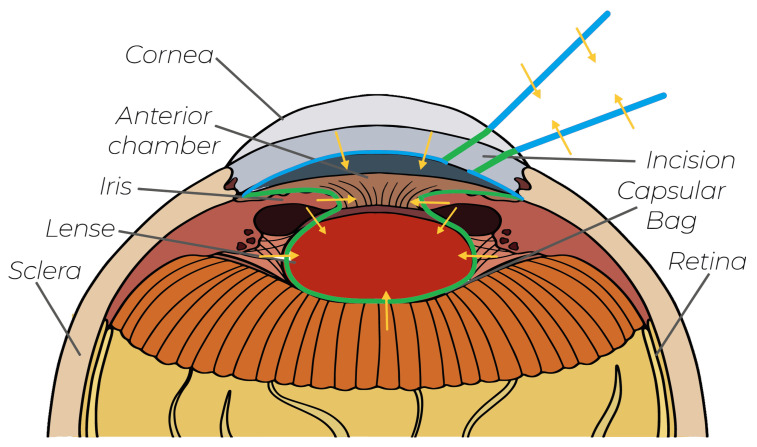
Existing concepts in cataract surgery: Green lines are the conventional virtual fixtures from the literature. The blue lines are our novel virtual fixtures, which are implemented in this work. The orange arrows indicate the FF directions.

Cataract removal typically demands several hours of training for young surgeons to achieve proficiency [13]. As an enhancement to the existing simulation-based training setups, a haptic human–machine interface could accelerate the learning process for novice surgeons, reducing the time required to reach competency [5]. To address this need, we propose a shared control system that incorporates virtual fixtures to effectively guide the surgeon’s actions. The contributions of this work can be summarized graphically by Figure 1: (1) virtual-fixture-based shared control for the incision and (2) virtual-fixture-based protection of the posterior corneal surface. Furthermore, a usability study is conducted, in which the benefits of our virtual-fixture-based shared control is validated.

The remainder of the paper is organized as follows: in Section 2, the state of the art and the research gap are discussed. Section 3 presents the technical system used for the development and validation of our shared control concepts. This is followed by the presentation of our novel virtual-fixture-based shared control guidance in Section 4. Then, our pilot study is presented in Section 5 and their results are discussed in Section 6. Finally, Section 7 concludes our work.

## 2. State of the Art and Research Gap

This state-of-the-art overview first includes the *Teleoperation and Shared Control Medical Assistance Systems* in a general manner, which is followed by the discussion of the *Cataract Surgery Training and Safety Systems*. Please note that no learning-based and black-box models or concepts are presented in this overview, since their formal admissions by the FDA or EMA (FDA: *Food and Drug Administration* and EMA: *European Medicines Agency*) are still challenging and open. However, they provide interesting and promising approaches.

As an add-on to a simulation-based training system like [14], an adaptive human–machine interface is a promising accelerator for young surgeons, reducing the required time to be able to conduct cataract operation on their own. To address this, we propose a shared control system that incorporates virtual fixtures to guide the surgeon’s actions, combining robotic assistance with human-in-the-loop characteristics; see [15]. Our proposed cooperative assistance system enhances surgical outcomes and facilitates faster, more effective training for new surgeons, providing a compelling solution for the future of surgical education and practice.

### 2.1. Teleoperation and Shared Control Medical Assistance Systems

In the literature, there are various shared control and teleoperation systems for robot-assisted surgeries for different types of operations; see, e.g., robot-assisted vitreoretinal surgery [16]. One of the most commonly used teleoperation systems is the Da Vinci, which has been used in clinical practice for minimally invasive surgeries and research worldwide since 2000 ([17] (Chapter 9)), ([18] (Chapter 3)). The integration of haptic feedback for teleoperating surgeons into the Da Vinci system was only realized in 2024 due to the challenges in designing intuitive feedback that meaningfully improves the system’s safety and efficiency; see [19,20].

The importance of a haptic FF for surgical teleoperation systems has been highlighted in [21], where the fundamental challenges of providing haptic feedback are discussed. Furthermore, many current robot-assisted surgical systems are at level 1 according to LASR (*Levels of Autonomy in Surgical Robotics*), as stated in [8,22]. Thus, in the near future, haptic support can also be beneficial for training since surgeons will not be completely replaced by automation in the next few years.

Such haptic support is realized by means of shared control systems [23], which involve “*sensory awareness and motor accuracy of the surgeon, thereby leading to improved surgical procedures and outcomes for patients*”. Such a shared control system is presented in [24,25], in which shared control systems are proposed for drilling craniotomy and mandibular angle split osteotomy robot-assisted surgeries. Both indicated that surgical procedures with haptic feedback had better outcomes compared to manually conducted ones.

In [26], dynamic virtual fixtures are generated from intra-operative 3D images, which are used to generate haptic feedback for the operating surgeon. The proposed method is used for thoracic surgeries, in which the main focus is the generation of *forbidden-region virtual fixture*; see [26] for more details. In [27], virtual fixtures are generated from a reference path, and are used as an attractive potential. In [28], a shared control system is presented for medical applications. Even though the authors describe their system as *shared control*, there is no simultaneous haptic interaction between automation and the human surgeon: specific subtasks of the bimanual peg transfer task are automated by trained, data-based control algorithms to optimally support the surgeon.

### 2.2. Cataract Surgery Training and Safety Systems

Unlike systems aimed at full automation, virtual fixtures are designed to enhance the operator’s medical proficiency, which is an advantageous characteristic for medical training systems. Since shorter cataract surgeries are associated with improved patient outcomes [29], it is crucial that young surgeons quickly achieve proficiency, as surgical experience significantly reduces operation duration [30].

In the current state-of-the-art training systems, there are approaches that help surgeons to reach the necessary experience level. An adaptive and skill-oriented training system is presented in [31], in which the benefits of a systematic training curriculum are validated in a feasibility study. Such systems could be extended in the future with our proposed haptic shared control concept. In [32], haptic guidance is proposed for the training of surgeons; however, it is based on the so-called Transformer model, which requires a predefined reference trajectory of the path. In our work, we have no reference: the FF is directly generated from the virtual fixtures.

To optimally challenge inexperienced surgeons, in [33], an *optimal-challenge-point* controller is proposed based on the skill assessment of the training surgeon. In [34], a Unity-based training simulator is proposed, which can be used for the virtual training of young surgeons. However, the system does not provide haptic feedback, which would be beneficial for reducing their training time. A recent thesis [35] was proposed using haptic cues, suggesting that guidance-based and error-amplifying haptic feedback training could improve performance, especially among participants with initially lower skill levels in robot-assisted telesurgery.

In the context of cataract operations, a few studies in the literature have explored the use of virtual fixtures for generating FF. In [9], a serial robot was employed to implement various types of virtual fixtures for teleoperated cataract surgery. These virtual fixtures included a remote center-of-motion control algorithm to protect the corneal incision site, as well as constraints to facilitate the safe removal of the lens. The virtual fixtures were designed to automatically adapt to eye rotations, providing protection to the posterior side of the iris and the capsular bag. This approach relied on geometric primitives derived from a mathematical eye model. Another method for incision protection is detailed in [36], where a cooperative robotic system was used to measure interaction forces between the surgical tool and the scleral incision. Various control algorithms were tested on a dynamic physical eye model to limit interaction forces, including a virtual fixture that constrained motion when the interaction force surpassed a predefined threshold. In [37], a torus-shaped virtual guidance force field was proposed to assist in capsulorhexis during cataract surgery, effectively preventing ruptures of the anterior capsular bag and damage to the interior of the iris.

### 2.3. Research Gap

In summary, virtual fixtures can effectively protect certain eye regions that are vulnerable to complications during cataract surgery, see Table 1. To further reduce the risk of complications in a future cataract surgery, complementary virtual fixtures are introduced in this paper. By integrating the proposed algorithms, simulation-based training systems can provide enhanced support for trainee surgeons. Thus, the contributions of this paper are the following:

- **Guidance to Incisions**: This paper uses virtual-fixture-based shared control to generate FF for the guidance of surgeons along a predefined axis to the incision point. The novel guidance concept incorporates an efficient geometrical representation of the virtual fixtures, which can be directly taken from the soft tissue geometry, making our concept generalizable.
- **Protection of the Posterior Corneal Surface**: The posterior corneal surface is fragile, and therefore it must not be touched during manipulation inside the anterior chamber. A semisphere-shaped virtual fixture is used to generate FF toward the center of the anterior chamber, guiding the operating tool away from the cornea.

**Table 1:** Literature on virtual fixtures in ophthalmic surgery. A checkmark (✓) indicates that a work solves the challenge, while a cross (✗) signifies a failure to do so.

Work	Positioning Support for Incision	Protection of Incision	Protection of Posterior Cornea	Protection of Iris	Guidance for Capsulorhexis	Protecting Capsular Bag
[9]	✗	✓	✗	✓ back side	✗	✓
[38]	✗	✓	✗	✗	✗	✗
[37]	✗	✗	✗	✓ inner side	✓	✗
[39]	✗	✓	✗	✗	✗	✗

To visually summarize the contributions of this work, these two novel protection areas are marked in purple in Figure 1. Additionally, an experimental setup was established, and a pilot study was conducted to validate and analyze the proposed incision-point-finding shared control method, since, in the literature, there has been no research work conducting such a study.

## 3. System Overview

**Figure 2:**
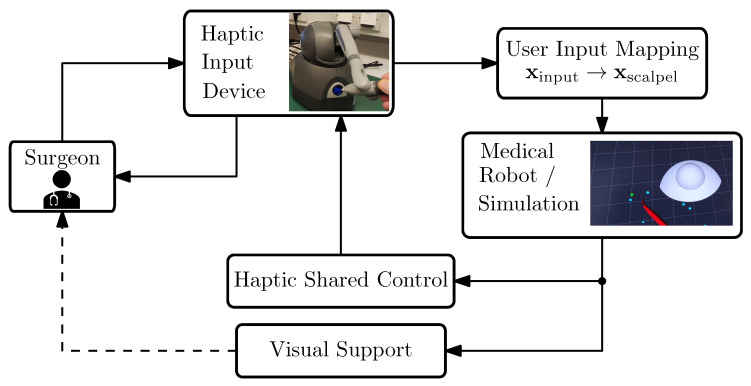
System overview of our robotic-assisted surgical system. The arrows indicate the communication flow within our system.

This section introduces the technical system used for the development and validation of our virtual-fixture-based shared control concept; see Figure 2 with the structure of the teleoperation surgery system. Furthermore, the requirements of the shared control system are discussed.

### 3.1. Technical System

Due to resource optimization, the current research utilizes a simulated environment for the medical robot. This decision does not negatively influence the validation or verification of our shared control concepts, as the accuracy of robot motion tracking in the simulation environment remains high.

In robot-assisted telesurgery, surgeons rely exclusively on visual and haptic interfaces to interact indirectly with patients, since the robot handles physical contact remotely. This separation results in the absence of direct feedback to surgeons, creating additional challenges; see, e.g., ([21], pp. 341–342). Our system works with the ROS2 middleware and includes the following components and functionalities:

- A haptic input device, a *3D Systems Touch* (in the literature, this device is also referred to as the master robot, haptic input device, etc. For brevity, in this work, we will refer to it simply as the *input device*. More information can be found on the manufacturer’s website and in e.g., [40]).
- Mapping of the user inputs to the medical robot’s motion.
- Simulation of the scalpel’s motion. In our framework, we used SOFA for soft tissue interaction while we employed RViz for rapid deployment in situations that did not demand detailed modeling of tissue–scalpel interactions.
- Haptic shared control function. In general, this function can be virtual-fixture-based, model-based, or model-free. In this work, we propose a virtual-fixture-based haptic support.
- Visual clues for the guidance. It has been shown that visual cues can be helpful for training inexperienced surgeons; therefore, our setup includes visual guidance as well; see [41].

Users operate the input device similarly to holding a pen, freely moving it within its workspace to control the teleoperated medical robot. This device uses a serial kinematic structure resembling a robotic arm, featuring six revolute joints. Positional FF is provided via actuation of the first three joints, though the underactuated structure prevents orientation FF (this is a common limitation in current teleoperation devices, as no commercially available input device offers full 6-DoF FF). The device offers FF of up to 3.3 N in any direction and incorporates two buttons at its end effector for extra functionality.

### 3.2. Technical Requirements

Based on discussions with our industrial partners and potential future users of the proposed shared control concept, the core requirements of the system are derived. These revealed that the following points should be respected for the implementation of virtual fixtures.

1. It must be safe at any time to release the input device. When operators relax their grasp, no dangerous motion should result from the generated FF.
2. The system should have a modular architecture. Different types of virtual fixtures should have the same interfaces to be easily exchangeable. This is necessary for the generalization of the shared control concept for various applications.
3. The system should have a low latency. The time delay between measuring a new pose and setting the corresponding force field should not be perceptible by the user. The latency of the system needs to be tested.

In the following sections, the proposed shared control concept is presented in detail.

## 4. The Virtual-Fixture-Based Shared Control Guidance

We propose a virtual-fixture-based shared control that only uses a geometric representation of the virtual fixtures and goals, enabling the generalization for different applications. The chosen concept for the geometric representation of virtual fixtures involves using volumetric primitives, which define regions with and without FF. Basic geometric primitives allow for efficient data storage and evaluation through closed-form methods [42]. Rotationally symmetric virtual fixtures closely approximate the shape of eye tissue structures, while more complex shapes can be formed by combining multiple fixtures, as demonstrated in [9]. Constraints are designed to be hollow to prevent sudden directional changes around their center, which can cause instability. Virtual tubes and cones create unconstrained spaces for free robotic movement, with FF applied outside these areas; see, e.g., [43]. Unlike the approach described in [43], our system treats the constraint’s volume as the feedback region. This implies that FF occurs only upon exceeding the constraint boundaries and is absent within the central area or completely outside the constraint.

**Figure 3:**
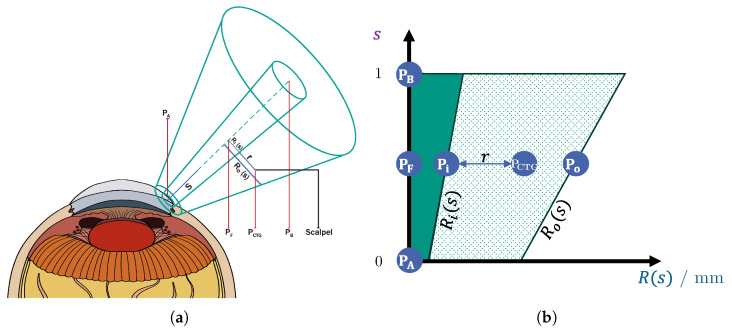
Sectional view of the constraint with a cone-shaped guide and its sectional perspective. (**a**) Cone-shaped virtual fixturefor shared control guidance. (**b**) Sectional view of the virtual fixture.

In this work, rotational symmetric constraints are used to guide the surgeon to the desired goal. An example of a rotational symmetric constraint can be seen in Figure 3b. It is used to guide surgical instruments toward the incision point. The geometric rotational symmetric constraints include the following parameters:

- Two points $(P_{A}$ and $P_{B})$ define the start and end of the rotational symmetric volume’s symmetry axis. The axis is parameterized with $s\in \left[0;1\right]$, so that s=0 at point A and s=1 at point B (see Figure 3).
- The inner and outer radius $\left(R_{i}\left(s\right)\mathrm{and}R_{o}\left(s\right)\right)$ being given as functions of *s*.

The rotational constraints are hollow: this means that their inner area around the axis has FF characteristics different from the outer part. This division helps to prevent sudden changes in FF generation; see Figure 3b. In our current implementation, there is no FF in the inner hollow part of the constraint; see the dark green area in Figure 3b.

Evaluating the rotational symmetric virtual fixtures involves two major steps: first, we find the perpendicular foot point ($P_{F}$) for the tool position ($P_{\mathrm{ctg}}$), meaning the location where the constrained tool geometry (ctg) projects onto the central axis of the fixture. If $P_{F}$ is not between points $P_{A}$ and $P_{B}$, the tool is outside the constraint. If it is between those points, the evaluation process is continued. To calculate the inner and outer radii of the fixtures at the tool point, the parameter $s\in \left[0;1\right]$ is computed. It is the axial distance of the foot point from point A relative to the height *h* of the constraint (1); see Figure 3a.

(1) $$s=\frac{\left|\right|P_{F}-P_{A}\left|\right|}{h}=\frac{\left|\right|P_{F}-P_{A}\left|\right|}{\left|\right|P_{B}-P_{A}\left|\right|}$$

Then, a point on the inner radius $P_{i}$ and a point on the outer radius $P_{o}$ are calculated based on *s* such that

(2) $$\begin{array}{cc} P_{i}\left(s\right) & =P_{F}+\left(P_{\mathrm{ctg}}-P_{F}\right)\cdot \frac{R_{i}\left(s\right)}{\left|\right|P_{\mathrm{ctg}}-P_{F}\left|\right|} \end{array}$$

(3) $$\begin{array}{cc} P_{o}\left(s\right) & =P_{F}+\left(P_{\mathrm{ctg}}-P_{F}\right)\cdot \frac{R_{o}\left(s\right)}{\left|\right|P_{\mathrm{ctg}}-P_{F}\left|\right|} \end{array}$$

The tool’s distance to the middle axis $d=\left|\right|P_{\mathrm{ctg}}-P_{F}\left|\right|$ and the two radii of the virtual fixtures, $P_{i}\left(s\right)$ and $P_{o}\left(s\right)$, are then compared to decide whether the tool is in the constraint’s hollow center, inside the constraint, or outside. Note that the concept is generalizable to semisphere constraints, in which the radii $R_{i}$ and $R_{o}$ are constant.

### 4.1. Goal Point and Gain Adaption

For the proposed virtual fixture-based shared control, the following are implemented:

- In the hollow middle cylinder, there is no FF, since here, the surgeon moves the tool into the right direction. Thus, this hollow-formed fixture definition helps to maintain a more intuitive motion.
- The inner radius is defined as the goal point, $P_{\mathrm{goal}}\left(s\right)=P_{i}\left(s\right)$, along which the tool is attracted.
- The outer radius defines the attraction zone, where the tool is pulled toward $P_{\mathrm{goal}}\left(s\right)$.

The rotational symmetric constraint’s evaluation method finds the closest points of the constraint to the tool: one on the inner $P_{i}$ and one on the outer radius $P_{o}$; see Figure 3b.

It is used to calculate the tool’s position relative to the constraint

(4) $$p_{\mathrm{rel}}\left(s\right)=P_{\mathrm{goal}}-P_{\mathrm{ctg}},$$

from which the FF for the guidance is generated.

For the calculation of the FF, the gain parameter $k_{\mathrm{ax}}$ is defined, where the gain can be defined as a function of *s* (1). This means it changes in the axial direction. In a non-constant case, $k_{\mathrm{ax}}\left(s\right)$ grows along the middle axis starting from point B (s=1) and it reaches its maximum value $k_{max}$ at $P_{A}$ (s=0). In our work, we compared three different functions:

(5) $$k_{\mathrm{ax}}\left(s\right)=k_{max}\cdot \left\{\begin{array}{cc} 1 & \mathrm{constant}\mathrm{gain}\mathrm{function}\\ \left(1-s\right) & \mathrm{linear}\mathrm{gain}\mathrm{function}\\ \left(1-s^{2}\right) & \mathrm{quadratic}\mathrm{gain}\mathrm{function}. \end{array}\right.$$

**Figure 4:**
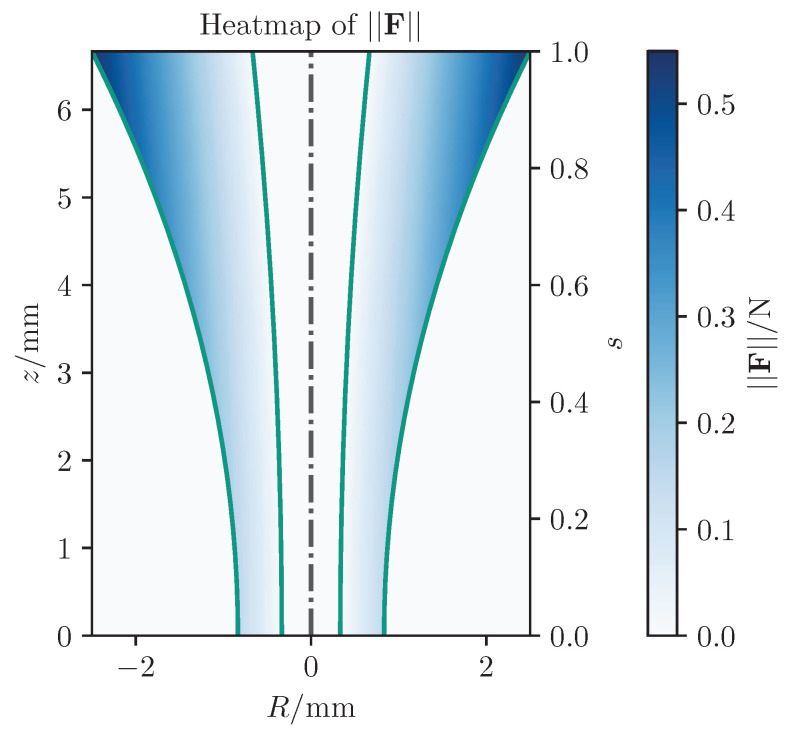
Heatmap of the absolute FF in sectional view of a rotational symmetric virtual fixture with a proportional feedback generation. The green lines represent the boundary defining the virtual fixture’s inner area.

A heatmap of the absolute forces for a virtual fixture with constant axial gain is given in Figure 4.

As illustrated in this figure, when the width of the attraction area is not considered, the maximum forces generated near the incision point during critical stages are insufficient for providing proper guidance to the surgeon. Therefore, gain adaptation is useful in scenarios where precise control is needed near the eye. As the tool approaches the eye, axial gain increases to maximize force around the incision point, ensuring accuracy. Farther away, weaker FF suffices since exact trajectories or points are less critical.

After calculating the gain, the FF is calculated using FF generation algorithms, which are described in the following subsection.

### 4.2. Force Feedback Generation

#### 4.2.1. Proportional Feedback Generation

**Figure 5:**
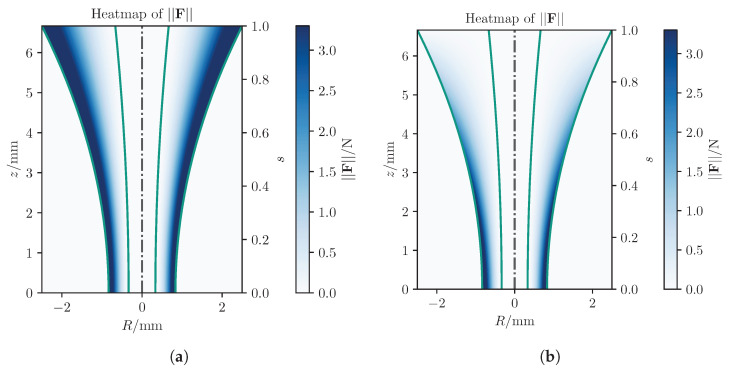
Heatmaps for the absolute force field calculated by a relative distance controller, in which the green lines represent the boundary defining the virtual fixture’s inner area. (**a**) Quadratic gain function in radial direction and constant axial gain. (**b**) Quadratic gain function in radial direction and linear gain function in axial direction.

In the state of the art of virtual fixture-based control methods, the usage of a proportional controller is commonly used. In that case, the FF is generated by

(6) $$F_{p}\left(s\right)=k_{\mathrm{ax}}\left(s\right)\cdot p_{\mathrm{rel}}\left(s\right),$$

where $k_{\mathrm{ax}}\left(s\right)$ depends only on the geometries of the defined constraints and the *s* coordinate. Rotational symmetric virtual fixtures can feature variable cross-sections, with inner and outer radii dependent on the parameter *s*, which helps to avoid unnatural changes in the FF generation. The FF along the axial direction is influenced by the radius, leading to a guidance toward $P_{A}$; see Figure 5b. Human proprioception is most comfortable when force grows roughly with the square of the error, as this keeps the specific mechanical work bounded; see [44]. Therefore, a quadratic gain offers both energetic consistency and intuitive haptic cues, enhancing surgical precision without causing abrupt or uncomfortable force changes.

Larger radii in the constraint lead to greater FF, whereas smaller radii produce much lower forces. This discrepancy makes it challenging to guide human surgeons toward their goals. Therefore, the FF at the end of the virtual fixture might not be strong enough and could be insufficient for guidance, see Figure 5. That issue is addressed in the following subsection.

#### 4.2.2. Relative Distance Feedback Generation

To address this challenge of insufficient FF, we propose novel relative distance FF generation in which the constant axial gain $k_{\mathrm{ax}}$ is modified and intensified by the radial gain $k_{\mathrm{rel}}$, leading to the FF generation

(7) $$F{\left(s\right)}_{p}=k_{\mathrm{ax}}\left(s\right)\cdot k_{\mathrm{rel}}\left(r_{\mathrm{rel}}\right)\cdot p_{\mathrm{rel},n}$$

where the relative distance and the unit normal vector are

(8) $$\begin{array}{cc} r_{\mathrm{rel}} & =\frac{\left|\right|p_{\mathrm{rel}}\left|\right|}{\left|\right|R_{o}\left(s\right)-R_{i}\left(s\right)\left|\right|} \end{array}$$

(9) $$\begin{array}{cc} p_{\mathrm{rel},n} & =\frac{1}{\left|\right|p_{\mathrm{rel}}\left|\right|}\cdot p_{\mathrm{rel}}, \end{array}$$

which are used to modify the FF for guidance. The axial gain $k_{\mathrm{rel}}\left(r_{\mathrm{rel}}\right)$ is defined as a function of the relative distance of $r_{\mathrm{rel}}$, such as

(10) $$r_{\mathrm{rel}}=\min\left[{\left(\frac{r_{\mathrm{rel}}}{r_{\max}}\right)}^{n},1\right],$$

where n=2. Note that different kinds of axial gain functions like tanh or exp could be realized. In this work, we do not investigate the impact of the different functions on the intuitiveness of the proposed shared control. Using (7), the benefits can be explained easily with Figure 5a,b: at the beginning of the motion, no strong FF is necessary since the surgeon is away from the soft tissue. Thus, a restriction is not necessary. However, moving closer to the eye, stronger guidance is necessary.

#### 4.2.3. Anisotropic Velocity Damping and Integral Feedback Generation

Since the input device is not physically connected to the scalpel of the medical robot, the damping of higher velocities can be calculated as follows:

(11) $$F_{\mathrm{damp}}=-d_{\mathrm{ax}}\left(s\right)\cdot v_{\mathrm{rel}},$$

where $d_{\mathrm{ax}}\left(s\right)$ is the damping factor depending on the distance from the incision point. It can lead to higher precision and more safety, since sudden changes are not allowed. Such virtual fixture-based controllers can also be found in the literature; see, e.g., [42]. However, using (11) might hinder the surgeon from performing tasks efficiently, since the generated FF always works against motions in every directions. This limitation of (11) leads to non-intuitive handling. Therefore, we introduce in this work anisotropic velocity damping FF generation, which overcomes this limitation. The core idea of the proposed anisotropic velocity damping is that it selectively penalizes motions opposing the intended direction of the virtual fixtures. Therefore, the proposed approach calculates the tool velocity in the desired direction:

(12) $$v_{\left|\right|}={\dot{P}}_{\mathrm{ctg}}\cdot p_{\mathrm{rel},\mathrm{norm}}={\dot{P}}_{\mathrm{ctg}}\cdot \frac{1}{\left|\right|P_{\mathrm{goal}}-P_{\mathrm{ctg}}\left|\right|}\left(P_{\mathrm{goal}}-P_{\mathrm{ctg}}\right)$$

Thus, the FF of the tool is modified, such as

(13) $$F_{\mathrm{damp}}=d_{\mathrm{ax}}\left(s\right)\cdot \left\{\begin{array}{cc} -v_{\|}\cdot p_{\mathrm{rel},\mathrm{norm}} & \mathrm{if}v_{\|}<0\\ 0 & \mathrm{if}v_{\|}\geq 0 \end{array}\right.$$

If the tool moves toward the goal point, no FF is applied. However, when the tool moves away from the goal point, FF is generated to resist that motion. This method ensures that only the velocity component opposing the desired direction is countered, while movements perpendicular or toward the goal point remain unimpeded by FF, leading to more intuitive interaction with the shared control.

Integrating the error over time ensures that the device reaches the target, even with minor deviations. Without this, the tool might stop short of the target. A possible solution could involve increasing the gain of the linear feedback in these nearby areas. However, finding the appropriate gains is challenging, time-consuming, and may result in aggressive, unintuitive feedback. To address this, an integral component is added such that

(14) $$F_{\mathrm{int}}=k_{\mathrm{int}}\left(s\right)\cdot \int_{t_{0}}^{t}p_{\mathrm{rel},n}\left(\tau \right)d\tau ,$$

where $k_{\mathrm{int}}\left(s\right)$ is a state-dependent integrator gain. $F_{\mathrm{int}}$ increases gradually, such that it ensures robustness against small deviations from the target.

#### 4.2.4. Lateral Filtering of the Feedback Generation

At the point where the surgeon interacts with the tool, the operating surgeon should receive tactile feedback from the tool–cornea interaction immediately upon reaching the incision point. This provides a realistic tactile response from the eye, enhancing the system’s credibility and acceptance among experienced surgeons. However, the superposition of virtual-fixture-based FF and physical contact forces occasionally results in non-intuitive undesirable outcomes within the shared control system, e.g., the generated forces guide the surgeon away from the optimal incision point.

**Figure 6:**
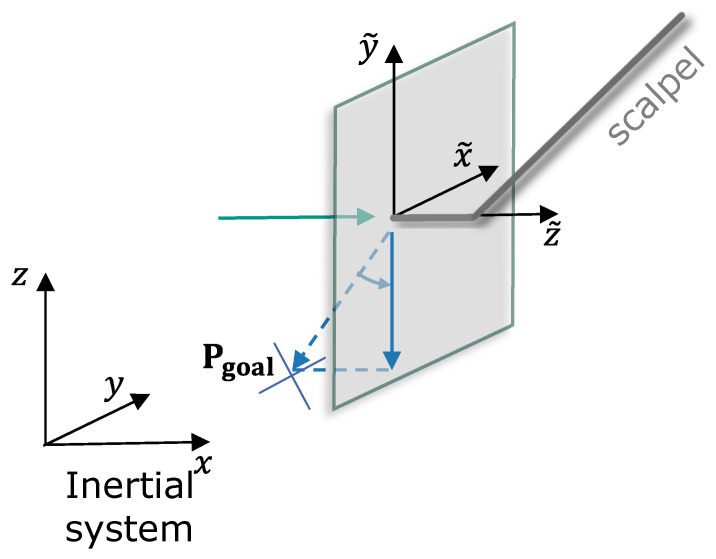
The principles of the proposed lateral filtering to obtain a goal-oriented feedback. The blue arrows show the decomposition of the force vector.

To avoid abrupt and sudden changes between the guidance and physical feedback, this work proposes lateral filtering: the physical FF only in longitudinal directions and the guidance virtual fixture-based FF only in lateral directions are transmitted. Its working principle is explained by Figure 6: the longitudinal vector is defined as parallel to the tool frame’s $\tilde{z}$-axis. On the other hand, the lateral vector is perpendicular to $\tilde{z}$. Thus, the FF vector is split up into lateral and longitudinal components:

(15) $$v_{\mathrm{longitudial}}=\left(v\cdot z_{\mathrm{ctg}}\right)\cdot z_{\mathrm{ctg}}$$

(16) $$v_{\mathrm{lateral}}=v-v_{\mathrm{longitudial}}$$

Only using the lateral component of a vector is, in this work, referred to as lateral filtering. It is not only used for the feedback force but also to project goal points of virtual force into the lateral plane (see Figure 6).

The vector v is the original vector representing the feedback force direction, which is decomposed into longitudinal (15) and lateral (16) components, showing how the force vector can be decomposed into a longitudinal (15) and a lateral (16) component. $z_{\mathrm{ctg}}$ is the unit vector of the tool frame’s z-axis in the inertial system. To summarize the feedback generation,

(17) $$F=F_{\mathrm{int}}+F_{\mathrm{damp}}+F_{p},$$

for which (16) is applied.

## 5. Initial Validation

To validate the usability of our novel virtual-fixture-based shared control concept, it is tested in a simulator setup.

### Validation Setup

**Figure 7:**
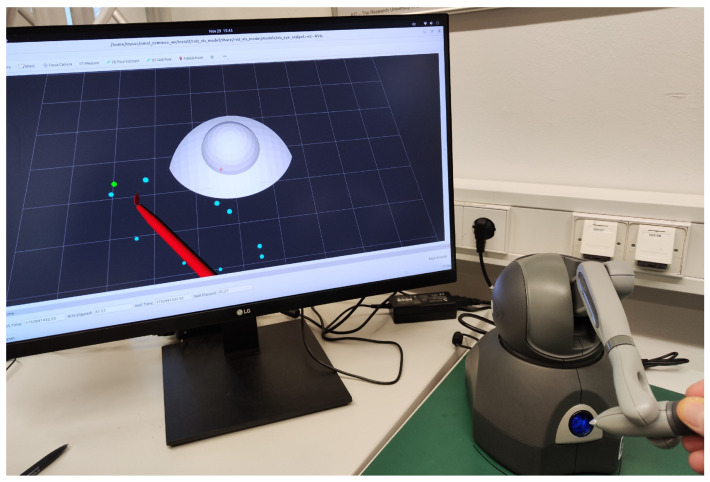
Illustration of the experimental setup featuring a haptic device used to control a virtual scalpel in our eye surgery simulation environment.

Figure 7 provides a visual illustration of the simulator configuration previously shown schematically in Figure 2. To streamline validation and concentrate solely on functional aspects, the simulation incorporates only the visual models of the eye and scalpel, thereby limiting external interference. It should be emphasized that achieving an accurate biomechanical simulation of corneal tissue interactions was not the objective, as the dynamics of soft tissue interactions were beyond this study’s scope.

Visual cues assisted the participant in performing the task, using an input device to control the scalpel. The incision start points were distributed in a circular area and initially highlighted in red. As the tool reached a point, its color changed to cyan, and completed tasks were marked in green. The task focused on both speed and positional stability at the target.

One participant performed the validation because we were unable to recruit enough qualified, active surgeons to conduct the experiment. The participant, who was not involved in the development, performed 12 incisions, each initiated from different randomized starting points. Three metrics were defined for performance evaluation:

1 Average completion time:
  (18) $$T^{\sum }=\frac{1}{N}\cdot \int_{t_{0}}^{t_{\mathrm{end}}}\tau d\tau ,$$
  where N=12 incisions. This metric evaluates the participant’s speed in completing the task.
2 Time spent near to the incision point (*critical proximity region*):
  (19) $$T_{\mathrm{near}}^{\sum }=\frac{1}{N}\cdot \int_{t_{0}}^{t_{\mathrm{end}}}\tau d\tau ,$$
  This metric assesses performance during the most critical phase regarding patient safety.
3 Average positional error within the critical proximity region:
  (20) e∑=1N·∫t0,neartendescalpel(τ)dτifescalpel(t)<ϵ  0else
  where $e_{\mathrm{scalpel}}\left(t\right)=∥x_{\mathrm{scalpel}}\left(t\right)-x_{\mathrm{inc}}∥$ and ϵ=30,mm define a safety-critical proximity.

We compared our virtual-fixture-based shared control concept with a classical potential field method using (6), where the axial gain is constant and does not depend on *s*. Since this gain is set to $k_{\mathrm{ax}}=1$, the feedback is only proportional to the distance from the center of the constraint. Furthermore, the case in which no haptic support was applied is taken into account.

## 6. Results and Discussions of the Initial Experiment

### 6.1. Results

The outcomes of the initial experiment are presented in Table 2. Our newly proposed virtual-fixture-based shared control method achieves an average completion time of 8.18 s, outperforming the standard potential field technique with constant feedback, which averages a 10.27 s completion time. Without haptic support, the participant required 11.49 s.

**Figure 8:**
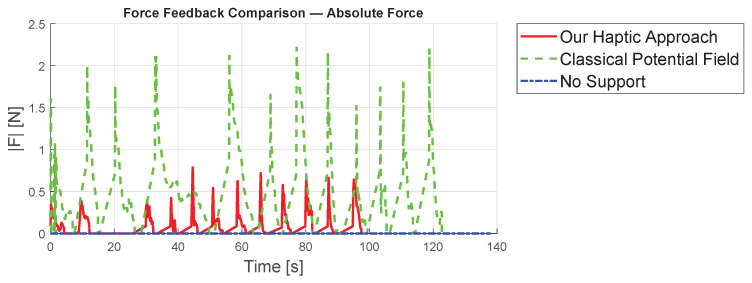
Comparison of the absolute value of the forces from the three different concepts.

**Figure 9:**
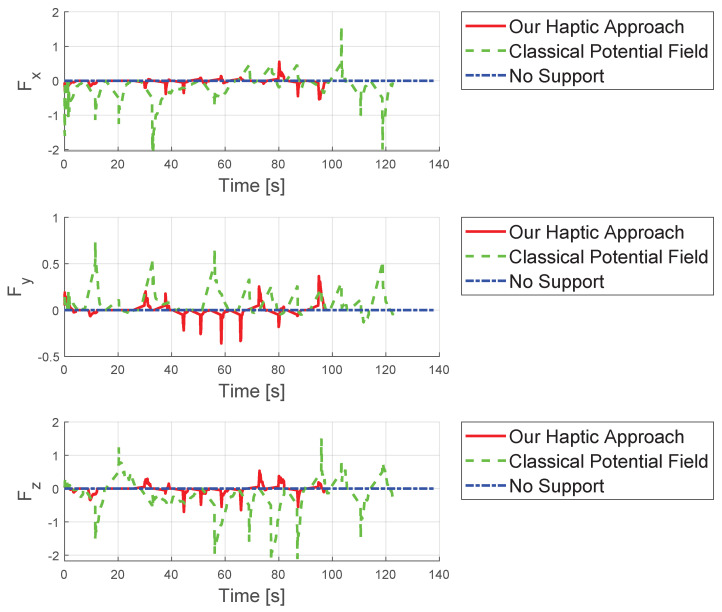
Comparison of the forces from the three different concepts evaluated in the three spatial directions.

Analyzing maximum forces as shown in Figure 8, the classical potential-field method exerts significantly larger forces, reaching up to app. 2.1 N, compared to 0.8 N with our approach, largely due to the relatively large initial distance. Figure 9 presents detailed forces along individual axes. In summary, the proposed concept leads to improved results compared to existing methods from standard techniques.

**Figure 10:**
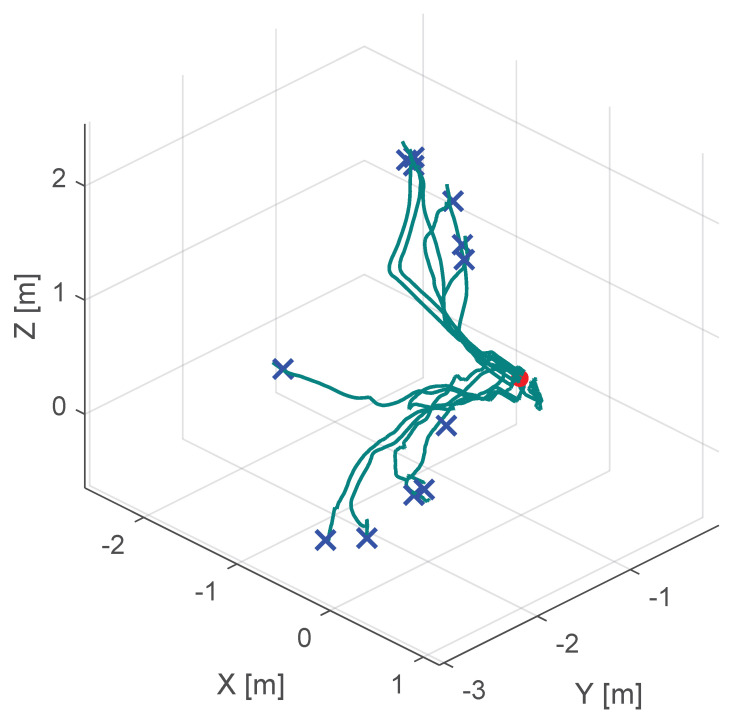
Course of the trajectory of the our novel virtual-fixture-based shared control concept. The goal point is the red dot, the blue crosses are the various starting points and the green lines are the resulting trajectories.

**Figure 11:**
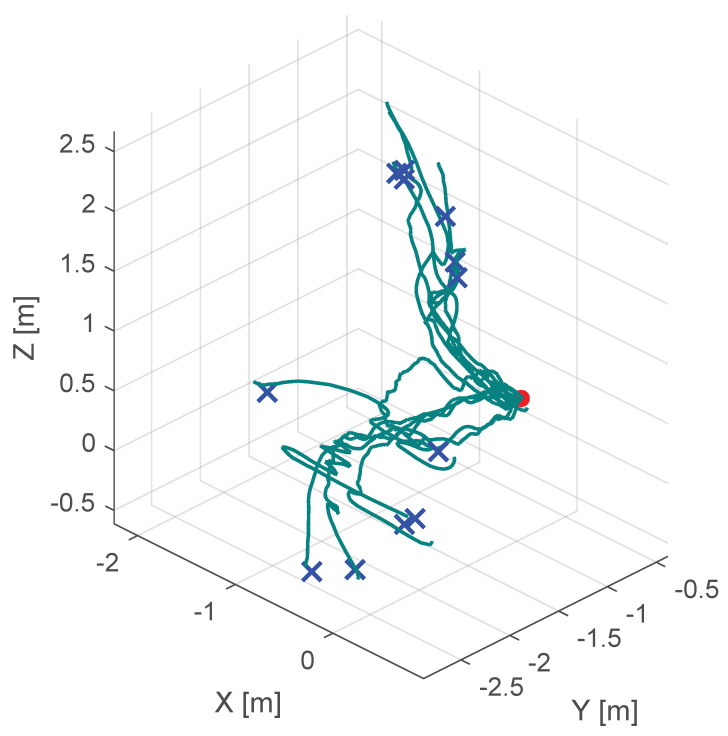
Course of the trajectory of the classical potential field haptic support. The goal point is the red dot, the blue crosses are the various starting points and the green lines are the resulting trajectories.

**Figure 12:**
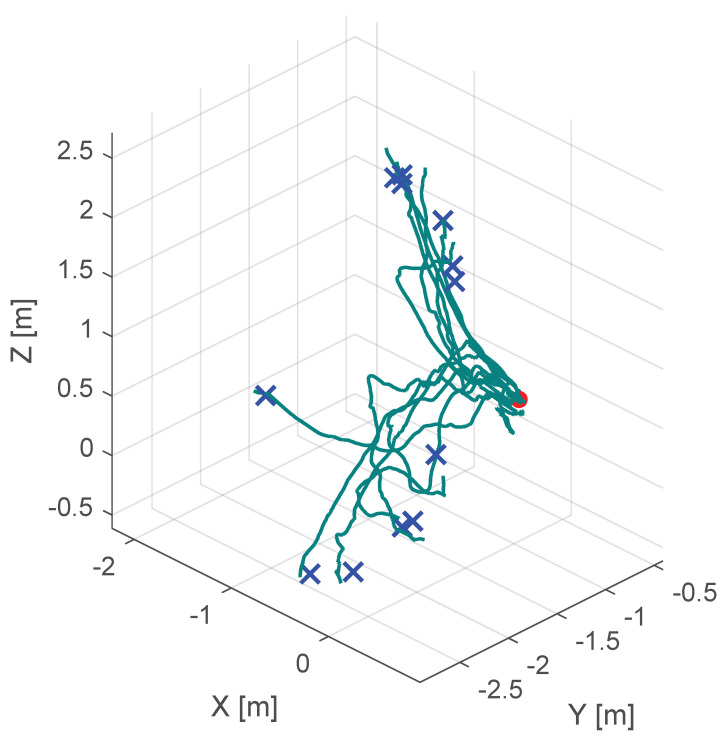
Course of the trajectory of the concept without haptic support. The goal point is the red dot, the blue crosses are the various starting points and the green lines are the resulting trajectories.

Additionally, Figure 10, Figure 11 and Figure 12 show the corresponding trajectories. As depicted in the center of Figure 11, the classical potential-field approach produces oscillations caused by its constant feedback gain. In contrast, the trajectory obtained with the proposed method is noticeably smoother, demonstrating that the operator remains closer to the primary cutting axis; see Figure 12. When no supportive feedback is applied (center of Figure 12), movement toward the target point becomes non-linear.

Thus, the proposed assistance system is particularly beneficial for training purposes, as younger physicians can thereby learn which movements best align with the desired main cutting point. Consequently, the method is well suited for integration into surgical simulators aimed at educating less experienced practitioners.

### 6.2. Limitation of the Current Experimental Setup

Despite promising initial outcomes, some limitations inherent in the experimental setup necessitate careful interpretation of our findings. Firstly, the experiment involved only a single participant who was not medically trained, which significantly restricts the generalizability and external validity of the results. Moreover, the simulation environment employed in this study was simplified, excluding realistic soft-tissue interactions such as deformation and realistic tactile responses, potentially reducing the relevance of the findings to real surgical scenarios. Additionally, the short-term nature of the validation prevents analysis of learning effects or long-term skill acquisition. Finally, the experimental setup did not include typical stressors present during actual surgeries, such as time pressure or patient-critical scenarios. Nevertheless, these initial findings show strong potential, and addressing these limitations thoroughly forms a central aspect of our planned future research activities.

## 7. Conclusions and Outlook

In this work, we propose a novel virtual-fixture-based method specifically designed to train young surgeons in performing cataract operations according to established standards. By implementing non-linear scaling of force feedback, our method effectively prevents large and potentially disruptive feedback forces, enhancing both safety and comfort for the trainee surgeon. The novelty of our approach lies in its geometric representation, which enables adaptability to various shapes and leads to a generalized formulation applicable to different surgical operations, thus facilitating broader training for surgeons throughout their professional careers. This generalization could significantly accelerate surgical training across various medical disciplines, addressing the global shortage of skilled surgeons, particularly in developing regions where advanced surgical expertise remains limited.

In the future, we plan to extend our framework to cover additional phases of cataract surgery, such as capsular bag tearing. Assisting with these challenging tasks within a shared control system promises to shorten the learning curve significantly, allowing novice surgeons to rapidly achieve proficiency, thereby increasing surgical precision, patient safety, and overall procedural success. Identifying novice surgeons [45] and implementing shared control systems that account for human variability (see, e.g., [46]) are essential for our future work to ensure both adaptability and safety.

## Figures and Tables

**Table 2:** Results of the initial validation.

	$T^{\sum }$	$T_{\mathrm{near}}^{\sum }$	$e^{\sum }$
No haptic support	11.49 s	2.54 s	20.31 mm
Classical potential-field-based shared control	10.27 s	1.12 s	19.22 mm
Our virtual-fixture-based shared control	8.18 s	0.41 s	17.11 mm

## Data Availability

The data presented in this study are available on request from the authors.

## Abbreviations

The following abbreviations are used in this manuscript:

FF	Force Feedback
FDA	Food and Drug Administration
EMA	European Medicines Agency
CTG	Constrained Tool Geometry
